# Production of Virus-Derived Ping-Pong-Dependent piRNA-like Small RNAs in the Mosquito Soma

**DOI:** 10.1371/journal.ppat.1002470

**Published:** 2012-01-05

**Authors:** Elaine M. Morazzani, Michael R. Wiley, Marta G. Murreddu, Zach N. Adelman, Kevin M. Myles

**Affiliations:** Department of Entomology, Fralin Life Science Institute, Virginia Tech, Blacksburg, Virginia, United States of America; University of California Riverside, United States of America

## Abstract

The natural maintenance cycles of many mosquito-borne pathogens require establishment of persistent non-lethal infections in the invertebrate host. The mechanism by which this occurs is not well understood, but we have previously shown that an antiviral response directed by small interfering RNAs (siRNAs) is important in modulating the pathogenesis of alphavirus infections in the mosquito. However, we report here that infection of mosquitoes with an alphavirus also triggers the production of another class of virus-derived small RNAs that exhibit many similarities to ping-pong-dependent piwi-interacting RNAs (piRNAs). However, unlike ping-pong-dependent piRNAs that have been described previously from repetitive elements or piRNA clusters, our work suggests production in the soma. We also present evidence that suggests virus-derived piRNA-like small RNAs are capable of modulating the pathogenesis of alphavirus infections in *dicer-2* null mutant mosquito cell lines defective in viral siRNA production. Overall, our results suggest that a non-canonical piRNA pathway is present in the soma of vector mosquitoes and may be acting redundantly to the siRNA pathway to target alphavirus replication.

## Author Summary

Mosquitoes defend themselves against viral infection with an innate immune response. Thus, mosquito-borne viral diseases like West Nile fever, dengue fever, and chikungunya fever are transmitted to humans only when the pathogen overcomes these defenses. Despite this, relatively little is known about the immune pathways of the mosquito. We have previously shown that an antiviral response directed by small interfering RNAs (siRNAs) is present in culicine mosquito vectors. However, we show here that another class of virus-derived small RNAs, exhibiting many similarities with ping-pong-dependent piwi-interacting RNAs (piRNAs), is also produced in the soma of culicine mosquitoes. We also show that these piRNA-like small RNAs are capable of mounting an antiviral defense in mosquito cell lines with defective siRNA-based immunity, suggesting that mosquitoes possess redundant RNA-based antiviral responses. This study provides new insights into how a mosquito's immune defenses restrict virus replication and the transmission of mosquito-borne viruses.

## Introduction

In plants and invertebrate animals the double stranded RNA (dsRNA) formed during the replication of RNA viruses is a potent inducer of an antiviral immune response directed by short interfering RNAs (siRNAs) [1]. In flies, exogenous dsRNA is processed by an RNase III enzyme, Dicer 2 (Dcr-2) [2]. The resulting siRNA duplexes (∼21 nt in length) are loaded into an RNA-induced silencing complex (RISC) [3]. These duplexes contain a guide strand that provides sequence specificity to the RISC, and a passenger strand that is removed from the activated RISC. The passenger strand is cleaved by Argonaute 2 (Ago-2), an essential component of the RISC possessing “slicer” activity [4]–[6]. Cleavage products are removed from the RISC by another endonuclease, C3PO [7]. The remaining guide strand directs the activated RISC to cognate RNAs in the cell, resulting in Ago-2-mediated cleavage and sequence specific degradation of the target molecules.

Although other small RNA pathways have not been ascribed an essential antiviral function, the recent identification of virus-derived Piwi-interacting RNAs (piRNAs; 25–30 nt in length) from a *Drosophila* ovary somatic sheet (OSS) cell line suggests an antiviral role for this pathway in the fly ovary [8]. Eukaryotic small RNAs can be distinguished from other non-coding RNAs in the cell by their associations with proteins in the Argonaute (Ago) -family. In *Drosophila*, a Piwi-clade is formed by the Ago proteins Piwi, Aubergine (Aub) and Ago-3. Antisense piRNAs have been found to associate with Piwi and Aub, while sense piRNAs associate with Ago-3 [9], [10]. Mutations in Piwi-clade Ago proteins have previously been shown to increase expression of transposable elements (TEs) in the fly ovaries [11], [12]. However, no clear role for this pathway has been established outside the germline. Ago-3 and Aub do not appear to be expressed in the *Drosophila* soma [9], [10], [13]–[15]. While a simplified, alternate version (Piwi-dependent, but Aub- and Ago-3-independent) of the piRNA pathway, called the primary pathway, has been demonstrated in the somatic cells that ensheath the ovary [12], [16], it remains unclear if piRNA pathways operate more broadly in the fly soma. Addressing this question has been complicated by the demonstration that piRNAs play a role in the epigenetic repression of transposable elements [17], [18]. A class of small RNA sequences 24–27 nt in length have been mapped to transposons in the soma of Ago-2 mutant flies [19]. Silencing tandem arrays of a *white* transgene in the eyes of wild type flies has also been shown to require functional Piwi and Aub [20]. However, it is not clear if these represent examples of somatic piRNA production or maternal inheritance of piRNA populations.

Evidence suggests that Dicer is not involved in the production of piRNAs [11]. Most piRNAs are derived from a few genomic loci known as piRNA clusters and tend to be asymmetrical, mapping to a single genomic strand in the piRNA cluster [9], [21], [22]. In contrast to the siRNA duplexes generated by Dicer, complementarity between sense and antisense piRNAs is generally limited to 10 nucleotides at the 5′ ends [9], [10]. This has led to a 'ping-pong' model of piRNA biogenesis [9], [10]. Many aspects of this model remain theoretical, having been deduced from small RNA profiling studies [9], [10]. In the model, piRNAs derived from a specific strand direct the production of piRNAs from the opposite strand. The cleavage of target strands directed by piRNAs bound to Ago-3 determines the 5′ ends of piRNAs bound by Piwi and Aub, and vice versa [10], [23]. Evidence for this comes from the characteristic U-bias at the 5′ end of piRNAs bound by Piwi and Aub, which correlates with enrichment for adenine at the 10^th^ position of piRNAs bound by Ago-3 [9], [10]. While both the factors responsible and the location for processing (nucleus or cytoplasm) of the primary piRNAs that initiate ping-pong amplification cycles are unknown, the precursor substrates are believed to be derived from long single-stranded RNAs transcribed from repetitive elements or piRNA clusters [21], [24], [25]. Evidence for this comes from experiments in which a P-element insertion in the *flamenco* piRNA cluster disrupted production of piRNAs more than 160 kb downstream of the insertion site [9].

Many mosquito-borne viruses are associated with human and animal diseases. Most of these viruses have RNA genomes and are classified in one of three genera: *alphavirus*, *flavivirus* or *orthobunyavirus*. Although virus infections of the vertebrate host are acute and often associated with disease, the maintenance of these pathogens in nature generally requires the establishment of a persistent non-lethal infection in the insect host. The mechanism by which this occurs is not well understood, but we have previously shown that an antiviral response directed by siRNAs is important to the pathogenic outcome of alphavirus infections in the mosquito [26]. Expression of the heterologous flock house virus (FHV) B2 protein from a recombinant alphavirus increased virus replication in mosquitoes resulting in dramatic mortality [26]. The B2 protein of FHV is a well characterized dsRNA-binding protein that interferes with RNA silencing by preventing the protein components of the siRNA pathway access to the dsRNA-by-products of virus replication [27]–[29]. Pathogenesis associated with infection of mosquitoes with recombinant alphaviruses expressing B2 proteins is similar to an enhanced disease phenotype that has been described in *dcr-2* null mutant flies infected with various RNA viruses [26], [30]–[32]. Interestingly, mortality is not observed when culicine mosquitoes depleted of Dcr-2 are infected with alphaviruses [33], [34]. This led us to speculate that these animals might have redundant antiviral defenses. To investigate this possibility, we undertook a comprehensive survey of virus-derived small RNAs in culicine mosquitoes and cells infected with chikungunya virus (CHIKV; Genus: *Alphavirus*). Our results indicate production of virus-derived ping-pong-dependent piRNA-like small RNAs in the mosquito soma. Similar piRNA-like viral small RNAs appear to direct an antiviral response capable of modulating alphavirus pathogenesis in mosquito cell lines defective for siRNA production. Collectively, this work supports a model in which functionally-redundant RNA-based immune pathways target alphavirus replication in the soma of disease vector mosquitoes.

## Results

### Production of virus-derived ping-pong-dependent piRNA-like small RNAs in the mosquito soma

**Figure 1:**
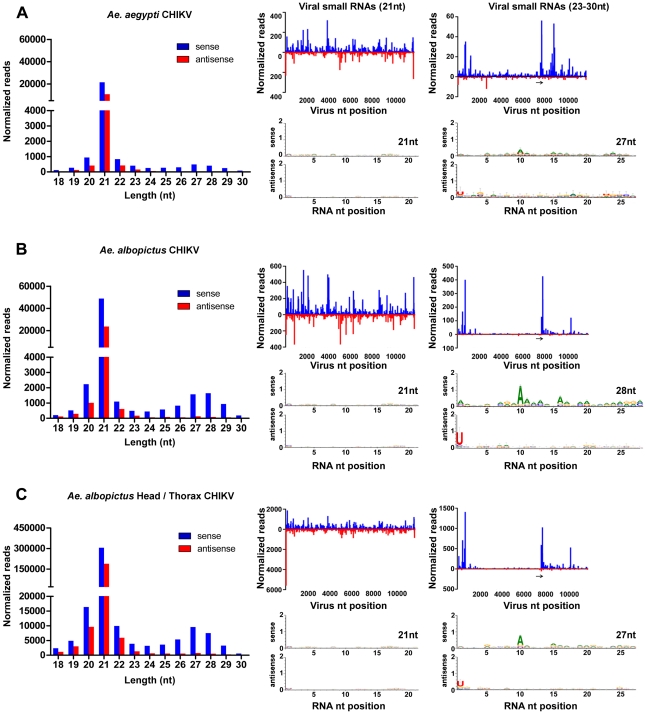
Production of piRNA-like viral small RNAs in the mosquito soma. Size distribution, density plots, and nucleotide analysis of virus-derived small RNAs in *A. aegypti* (**A**), *A. albopictus* (**B**) and head and thorax of *A. albopictus* (**C**) infected with CHIKV. Example weblogos are shown for the predominant size classes. Arrows denote approximate start of 26S mRNA.

To investigate the small RNA populations present in *Aedes aegypti* and *Aedes albopictus* mosquitoes infected with CHIKV, we prepared cDNA libraries from the ∼18–35-nt fraction of total RNA and sequenced on an Illumina platform. A detailed analysis of the sequenced virus-derived small RNAs in each library is presented in Table S1. As expected, viral siRNAs were the predominant small RNA species matching the CHIKV genome in both mosquitoes (Figure 1A and B). In the infected *A. aegypti* ∼1% of the total sequenced small RNAs were derived from the virus, while in the infected *A. albopictus* ∼1.5% of total sequenced small RNAs were of viral origin. In each of the infected mosquito species virus replication positively correlated with the accumulation of viral siRNAs, with higher levels of viral RNA resulting in more abundant viral siRNAs (Figure 1A, B and S1). Despite the observed differences in virus replication, production of virus was similar in both mosquito species suggesting that the greater accumulation of viral siRNAs in *A. albopictus* resulted in more robust modulation of the virus infection (Figure S1).

The first indication that the infected mosquito species also produced an abundant class of piRNA-like viral small RNAs was the presence of broad peaks ranging from 23–30-nt in the size distributions of reads mapping to the viral genome in each of the respective small RNA libraries (Figure 1A and B). Imbalanced synthesis of (+) and (−) strands is common among RNA viruses, with genomic strands typically produced in greater abundance than their counterparts. The genomic (+) strand (49S) RNA of an alphavirus serves both as mRNA and as a template for the synthesis of complementary (−) strand RNA. Viral (−) strands then serve as templates for the synthesis of new genomic-length (+) strand RNAs, as well as for shorter subgenomic (+) strand (26S) RNAs that encode the virus' structural genes. We have previously shown that alphavirus-derived siRNAs in infected mosquitoes do not cluster from any particular region of the virus genome, and are generated from (+) and (−) strands in proportions that suggest viral dsRNA replicative intermediates (RIs) are the primary Dcr-2 substrates for siRNA biogenesis [26], [35]. In contrast, 23–30-nt viral small RNAs in the infected mosquito species clustered near the 5′ termini of the genomic and subgenomic (+) strands, and consistent with previous descriptions of asymmetry in piRNAs derived from TEs were almost exclusively derived from virus (+) strands, suggesting biogenesis from the 49S and 26S mRNAs (Figure 1A and B).

Distinct piRNA pathways operate in the germline and somatic compartments of the *Drosophila* ovary [12], [16]. In the germline, piRNAs exhibit a bias for a 5′ uridine or an adenine at nucleotide position 10 when derived from the opposite strand, suggesting secondary amplification by a ping-pong-dependent mechanism [9], [10]. Previous descriptions of viral piRNAs in *Drosophila* OSS cells [8] indicate a strong preference for a 5′ U in the almost exclusively (+) sense reads, but no bias at the 10^th^ nt position in reads from the opposite strand, suggesting that these are products of a ping-pong-independent primary pathway [12], [15], [16]. While 5′ U and A10 signatures in the 23–30-nt viral small RNAs (Figure 1A and B) from *A. aegypti* and *A. albopictus* mosquitoes infected with CHIKV suggested biogenesis by a ping-pong-dependent pathway, the source (germline or soma) of these remained unclear as small RNA libraries were prepared from whole mosquitoes (Figure 1A and B).

Thus, to determine the source for production of piRNA-like viral small RNAs, we sequenced small RNA populations from the head and thorax (no ovaries) of *A. albopictus* infected with CHIKV. We observed a 5.7-fold enrichment of viral small RNAs (23–30-nt) exhibiting a bias for 5′ U and A10 signatures in mosquitoes lacking any ovarian tissues, suggesting that these were products of a ping-pong-dependant pathway present in the soma (Figure 1B and C). Specific analysis of sequenced pairs with a 10-nt offset in the predominant 27-nt size class indicated a significant enrichment (p-value <0.01; Adjusted Wald Test) for those with a 5′ U in the antisense read, and an A at the 10^th^ position of the sense read (example pair shown in Figure S1), providing additional support for ping-ponging in the soma. As the ping-pong-dependent piRNA pathway that has previously been described in *Drosophila* appears restricted to the germline [12], [16], our results suggest the 23–30-nt virus-derived small RNAs observed in the soma of mosquitoes infected with CHIKV are products of a non-canonical piRNA pathway.

Phylogenetic analysis of dipteran Ago proteins indicates a large expansion in the Piwi-clade since the divergence of *A. aegypti* and *Drosophila* [36]. So we next performed high throughput RNA sequencing (RNA-Seq) on the head and thorax of *A. albopictus* infected with CHIKV, in order to determine which of these genes are expressed in the soma of infected mosquitoes. In marked contrast to the Piwi-clade Ago proteins of *Drosophila,* we found transcripts from the entire repertoire of Piwi-subfamily genes (Ago-3 and 7 different Piwis) expressed in the soma of infected mosquitoes (data not shown).

### Increased production of virus-derived piRNA-like small RNAs during pathogenic infections

**Figure 2:**
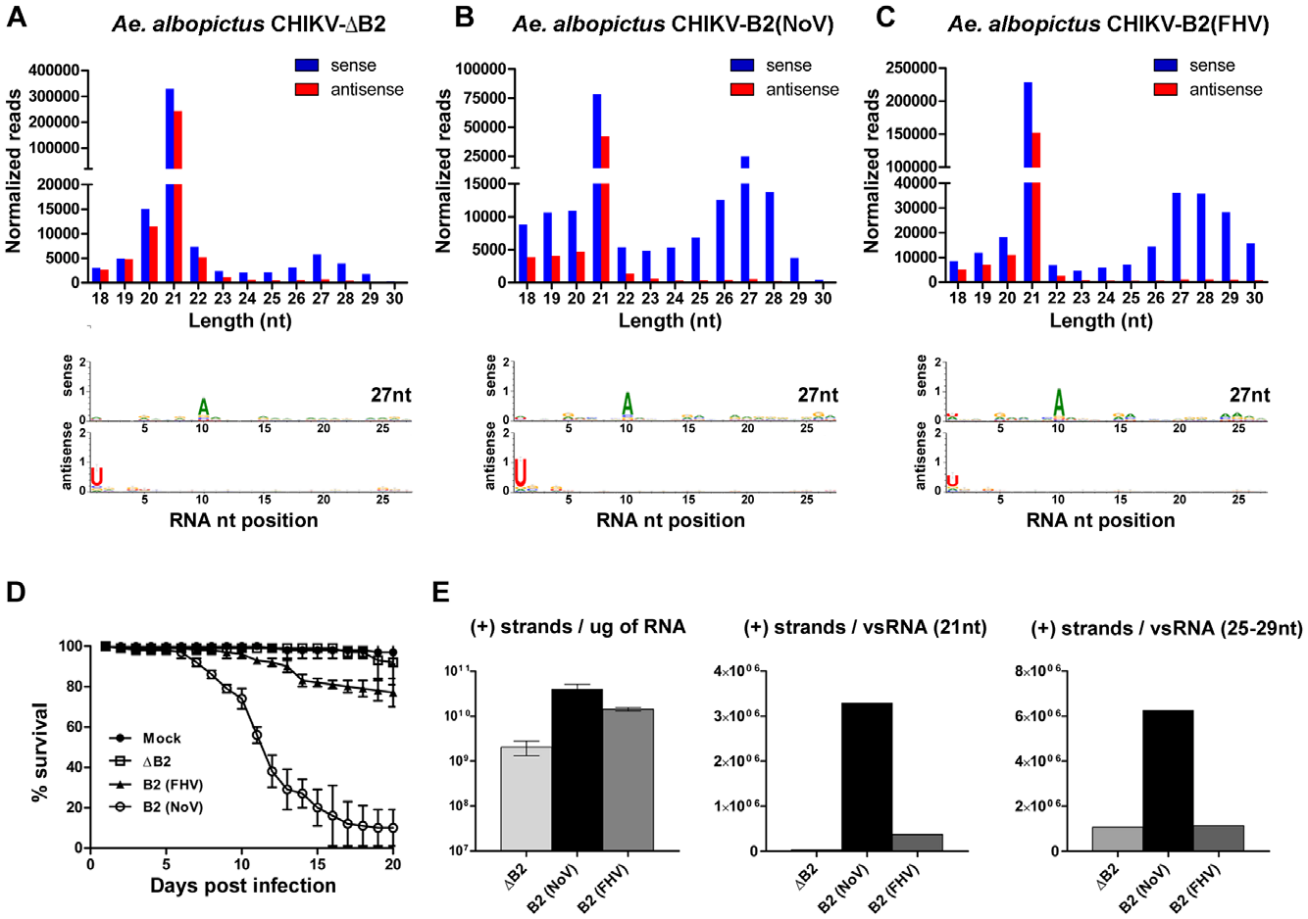
Production of piRNA-like viral small RNAs increases during pathogenic virus infections. Size distribution and nucleotide analysis of virus-derived small RNAs in the head and thorax of *A. albopictus* infected with CHIKV-ΔB2 (**A**), CHIKV-B2 (NoV) (**B**), or CHIKV-B2 (FHV) (**C**). Survival of *A. albopictus* after infection with recombinant CHIK viruses and mock injection (**D**). Error bars indicate the standard deviation among triplicate cohorts (n = 90). Strand-specific quantitative real-time PCR analysis of CHIKV (+) strands; shown as the number of copies per virus-derived small RNA in 1ug of total RNA (calculated from normalized reads identified in the corresponding library) (**E**). Error bars indicate the standard deviation among three biological replicates.

We next examined the production of piRNA-like viral small RNAs during pathogenic infections of *A. albopictus.* Mosquitoes were infected with a recombinant CHIKV expressing either the Nodamura virus (NoV) B2 or FHV B2 under the control of a second subgenomic promoter. A double subgenomic CHIKV containing an untranslatable NoV B2 (ΔB2; B2^-^B1^-^) that has been described previously [37] served as a control virus. As expected, expression of B2 (NoV or FHV) by CHIKV significantly decreased production of viral siRNAs in comparison to levels in mosquitoes infected with the control virus (p−value = 0; Figure 2A, B and C). However, suppression of viral siRNAs was greater when NoV B2 was expressed than during expression of FHV B2 (p−value = 0; Figure 2B and C). Analysis of piRNA-like viral small RNAs revealed increased production in mosquitoes infected with recombinant viruses expressing B2 proteins (p−value = 0; Figure 2A, B and C). Although increased mortality was observed in mosquitoes infected with recombinant viruses expressing B2 (NoV or FHV), infection with CHIKV-B2 (FHV) was less virulent than infection with CHIKV-B2 (NoV) (Figure 2D). Intriguingly, the less virulent phenotype of CHIKV-B2 (FHV) was associated with higher levels of viral siRNAs and piRNA-like viral small RNAs, when compared to levels in mosquitoes infected with recombinant viruses expressing the more potent NoV B2 (p−value = 0; Figure 2B and C). Although suppression of viral siRNAs by FHV B2 resulted in significantly higher levels of mRNA (p−value <0.01; Figure 2E), the average number of virus (+) strands per piRNA-like small RNA derived from CHIKV-B2 (FHV) was similar to that of the control virus (Figure 2E). This suggested that production of piRNA-like viral small RNAs increased in response to higher levels of viral mRNA in the infected mosquitoes. However, the correlation was not as apparent in the presence of a stronger NoV B2 suppressor protein, as the average number of virus (+) strands per piRNA-like small RNA derived from CHIKV-B2 (NoV) was higher than that of the control virus (Figure 2E). Although we cannot conclude from these results a role for piRNA-like viral small RNAs in restricting virus replication in the mosquito, experiments infecting *dcr-2* null mutants (currently unavailable) with alphaviruses are likely to be more definitive in this regard.

### Identification of *dcr-2* null mutant mosquito cell lines

We sequenced and analyzed small RNA populations from three *A. albopictus* (C6/36, u4.4, and C7-10) and two *A. aegypti* (Aag-2, and CCL-125) continuous cell lines infected with CHIKV. Aag-2 cells originated from embryos [38], while the CCL-125, C6/36, u4.4, and C7-10 cells all originated from homogenized mosquito larvae [39]–[42]. The CCL-125 and Aag-2 cell lines are non-clonal populations, and likely represent an amalgamation of various embryonic or larval tissue types [38], [42]. While the C6/36, u4.4, and C7-10 cell lines are subclones, the specific tissues from which they were generated are unknown [40], [41], [43].

**Figure 3:**
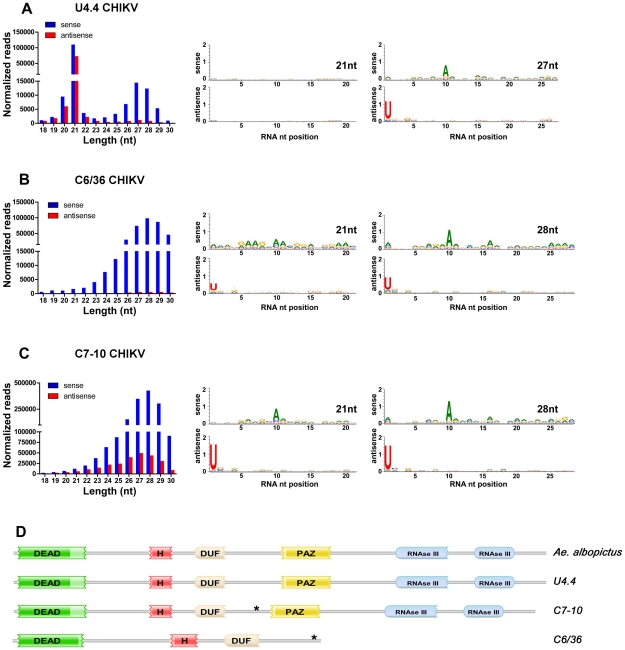
Identification of *dcr-2* null mutant mosquito cell lines. Size distribution and nucleotide analysis of virus-derived small RNAs in u4.4 cells (**A**), *dcr-2^FS−1^* (C6/36) cells (**B**) and *dcr-2^del 33^* (C7-10) cells (**C**) infected with CHIKV. Schematics indicating Dcr-2 domains (**D**). The *A. albopictus* Dcr-2 contains a DExH/D protein family domain (DEAD) and helicase conserved C-terminal domain (H); a domain of unknown function (DUF); a PAZ domain; and tandem RNase III domains. Asterisks indicate locations of deletions in *dcr-2* sequences.

We identified 21-nt small RNAs derived from CHIKV in each of the mosquito cell lines examined (Figure 3A, B, C and S2). However, the 21-nt size class was much less prominent in libraries prepared from the C6/36 and C7-10 cells, than in those prepared from u4.4, Aag2 and CCL-125 cells (Figure 3A, B, C and S2). A prominent class of 23–30-nt small RNAs derived from the virus was also evident in each of the cell lines examined (Figure 3A, B, C and S2). The clustering and asymmetry of these small RNAs suggested they were similar to the piRNA-like viral small RNAs identified in the soma of *A. albopictus* (Figure 1), but given the indeterminate origin of the cell lines it also remained possible they were analogous to a primary class of viral piRNAs previously described in *Drosophila* OSS cells [8]. However, nucleotide analysis of piRNA-like viral small RNAs identified in the mosquito cell lines revealed strong 5′ U and A10 signatures (Figure 3A, B, C and S2), suggesting biogenesis by a ping-pong-dependent pathway rather than the previously described primary pathway [12], [15], [16].

In contrast to the other cells used in this study, C6/36 and C7-10 cells lacked a prominent 21-nt peak in small RNAs derived from the virus (Figure 3A, B, C and S2). Analysis of the much less abundant 21-nt size class in these cells revealed clustering, asymmetry, and ping-pong signatures not present in the same size reads from any other cell line (Figure 3A, B, C and S2). This suggested that the 21-nt small RNAs derived from the virus in C6/36 and C7-10 cells were not siRNAs, but rather a subset of piRNA-like viral small RNAs (Figure 3A, B, C and S2). So we next sequenced the complete open reading frames (ORFs) encoding the Dcr-2 proteins of *A. albopictus,* u4.4, C6/36 and C7-10 cells. The *A. albopictus* Dcr-2 contained a DExH-box domain, a domain of unknown function (DUF), a PAZ domain, and two RNase III domains (Figure 3D). The u4.4 Dcr-2 differed from the *A. albopictus* sequence at only two conservative amino acid substitutions. In contrast, the C7-10 Dcr-2 contained an in-frame deletion of 33 amino acids (del 33) between the DUF and PAZ domains (Figure 3D). Genotyping determined that this deletion was homozygous, which is consistent with the apparent *dcr-2* null phenotype of these cells. Although the 33 amino acid indel does not occur within any identifiable domain structure, it may prevent the protein from assuming a functional three-dimensional configuration. Genotyping a single nucleotide deletion identified in the C6/36 *dcr-2* ORF revealed a homozygous frameshift mutation (FS −1) resulting in a premature termination codon. The predicted 821 amino acid protein lacks a portion of the PAZ domain and both RNAse III domains, which is also consistent with the apparent *dcr-2* null phenotype of these cells (Figure 3D). Taken together, these data suggest that the homozygous deletions identified in the C7-10 and C6/36 *dcr-2* alleles are null mutations that abrogate viral siRNA production in these cells. Additional details regarding the characterization of the continuous cell lines used in this study are provided in Text S1.

### Virus-derived piRNA-like small RNAs direct an antiviral response that modulates alphavirus pathogenesis in *dcr-2* null mutant mosquito cells

**Figure 4:**
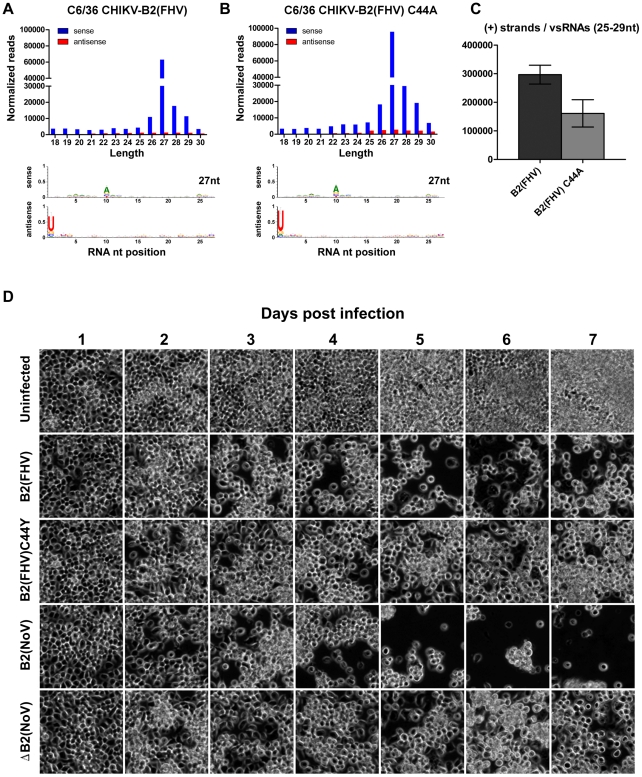
B2-mediated suppression of piRNA-like viral small RNAs in *dcr-2* null mutant cells. Size distribution and nucleotide analysis of virus-derived small RNAs in *dcr-2^FS−1^* cells infected with CHIKV-B2 (FHV) (**A**) or CHIKV-B2 (C44A) (**B**). Single representative TruSeq libraries are shown (replicate #2 in Table S1). CHIKV (+) strands per virus-derived small RNA in 1 ug of total RNA (calculated from normalized 25–29 nt reads identified in replicate TruSeq libraries) (**C**). Error bars indicate the standard deviation among three biological replicates. Modulation of alphavirus infection by an antiviral piwi-like RNA pathway in *dcr-2^FS−1^* (C6/36) cells (**D**). Time course of cytopathology in *dcr-2^FS−1^* (C6/36) cells infected with recombinant CHIK viruses (20X magnification).

We previously demonstrated severe cytopathology in C6/36 cells infected with a recombinant Sindbis virus (SINV; the prototype alphavirus) expressing FHV B2 [26]. Similar cytopathological changes were not observed in cells infected with a recombinant SINV expressing a C44Y FHV B2 mutant protein or with wild-type SINV [26]. The C44 residue of FHV B2 is important to the protein's suppressor activity due to its role in binding dsRNA [28]. Mutant C44A and C44S B2 proteins exhibit reduced dsRNA binding affinity [28], and the C44Y B2 is associated with decreased suppressor activity [26]. To determine if piRNA-like viral small RNAs present in *dcr-2^FS−1^* (C6/36) cells were specifically suppressed by FHV B2, we infected *dcr-2^FS−1^* (C6/36) cells with recombinant viruses expressing either the FHV B2 or C44A B2 and sequenced small RNA populations. Levels of viral piwi-like small RNAs were significantly lower in *dcr-2^FS−1^* (C6/36) cells infected with CHIKV-B2 (FHV) than in those infected with CHIKV-B2 (C44A) (p−value <0.01; Figure 4A and B), suggesting at least partial suppression of piRNA-like viral small RNAs by FHV B2. A significant increase in the number of CHIKV-B2 (FHV) (+) strands per piRNA-like viral small RNA relative to the control virus was also consistent with suppression by B2 (p−value = 0.02; Figure 4C). Although the average fold change in levels of piRNA-like viral small RNAs between the two experimental groups was relatively small, we were unable to distinguish between suppression of secondary products and suppression of primary products, which presumably initiate ping-pong amplification. Thus, rising levels of viral mRNA may have increased ping-pong amplification of secondary products, obscuring more robust suppression of primary piRNA-like viral small RNAs at this time point. To confirm that the suppression of piRNA-like viral small RNAs observed in the *dcr-2* null mutant mosquito cells was mediated by B2, we infected *dcr-2^FS−1^* (C6/36) cells with recombinant viruses expressing two other mutant FHV B2 proteins (C44Y and R54Q). In contrast to previous results with SINV-B2 (C44Y), infection of *dcr-2^FS−1^* (C6/36) cells with CHIKV-B2 (C44Y) caused mild non-lethal cytopathology, from which the cells ultimately recovered (Figure 4D). Importantly, more severe cytopathological changes were observed during infection with CHIKV-B2 (FHV) (Figure 4D). This phenotype correlated with decreased production of piRNA-like viral small RNAs (p−value = 0; Figure S3), and an increased number of virus (+) strands per piRNA-like viral small RNA four days after infection (Figure S3). An R54Q mutation in the FHV B2 has previously been shown to completely abolish the protein's ability to bind long dsRNA [27]. Similar to the results obtained with CHIKV-B2 (C44A and C44Y), infection of *dcr-2^FS−1^* (C6/36) cells with recombinant CHIKV expressing an R54Q mutant abolished the inhibitory effect of FHV B2 on the production of piRNA-like viral small RNAs (p−value = 0; Figure S3), suggesting that dsRNA-binding is essential to the suppressor activity of FHV B2 in these cells. Consistent with the weaker suppressor activity of FHV B2, infection with CHIKV-B2 (FHV) did not result in complete destruction of the cell monolayer. Therefore, we infected *dcr-2^FS−1^* (C6/36) cells with recombinant CHIKV expressing the stronger NoV B2 suppressor. A recombinant virus containing the untranslatable ΔB2 served as a control. Although we again observed mild cytopathology in cells infected with CHIKV-ΔB2, infection with recombinant virus expressing the NoV B2 suppressor resulted in more extensive destruction of the cell monolayer (Figure 4D). Similar results were obtained by infecting *dcr-2^del 33^* (C7-10) cells with CHIKV-B2 (NoV) and CHIKV-ΔB2 (Figure S3). Taken together, these data suggest a role for piRNA-like viral small RNAs in modulating alphavirus pathogenesis in *dcr-2* null mutant mosquito cell lines.

## Discussion

Antiviral pathways initiated by viral RNA have been shown in a diverse array of organisms to require amplification in order to mount an effective immune response. For example, effective RNA-based antiviral immunity in *C. elegans* requires amplification of the viral siRNAs generated from dsRNA RIs by an endogenous RNA-dependent RNA polymerase (RdRP) activity [1]. Similarly, some plants are thought to require RdRP-dependant production of secondary siRNAs in order to mount an effective antiviral immune response [44], [45]. However, RdRP-dependant production of secondary viral siRNAs has not been demonstrated in flies. In this study, we used massively parallel sequencing to survey small RNA populations in mosquitoes and cells from two different culicine species infected with CHIKV. In addition to 21-nt viral siRNAs, our analysis revealed widespread and abundant production of virus-derived piwi-like RNAs in the mosquito soma. However, in contrast to viral piRNAs previously described in an OSS cell line [8], piwi-like RNAs reported here contain a preference for a 5′ uridine or an adenine at the 10^th^ nucleotide position, depending on the virus strand from which they derive, suggesting amplification by a ping-pong-dependant mechanism.

We have previously shown that an RNA silencing response directed by 21-nt siRNAs modulates the pathogenic effects of alphavirus infection in the mosquito vector [26]. However, evidence presented here suggests that the pathogenesis of alphavirus infections in *dcr-2* null mutant mosquito cell lines, defective in viral siRNA production, is modulated by an antiviral immune response that is directed by piRNA-like viral small RNAs. Similarities between the piRNA-like viral small RNAs directing an antiviral immune response in *dcr-2* null mutant mosquito cells and those identified in the soma of *A. albopictus* suggests biogenesis by a conserved ping-pong-dependant pathway. It has been proposed that TE-derived piRNAs are produced from long, single-stranded RNA precursors transcribed from piRNA clusters [21], [24], [25]. However, our work in C6/36 cells suggests dsRNA is a precursor substrate in the biogenesis of piRNA-like viral small RNAs in the *dcr-2* null mutant mosquito cell line. Previous analysis of Argonaute proteins in dipterans indicates a large expansion in the Piwi-clade since the divergence of *A. aegypti* and *Drosophila* [36]. RNA-seq performed on the head and thorax of *A. albopictus* infected with CHIKV indicated somatic expression of transcripts from the entire repertoire of Piwi-subfamily genes. This is in marked contrast to the *Drosophila* soma where two of three Piwi-clade Ago proteins (Ago-3 and Aub) do not appear to be expressed [9], [10], [13]–[15]. Taken together, these results suggest the presence of a non-canonical piRNA pathway in the soma of vector mosquitoes that may be acting redundantly to an antiviral immune response directed by siRNAs.

Imbalanced synthesis of (−) and (+) strands is a common feature of RNA viruses, typically with production of the genomic strand being greater than its full-length complement. Alphaviruses are thought to synthesize (−) strand RNAs for a limited duration of time early in the infection [46], presumably establishing an upper limit on the number of dsRNA RIs present in the cell. However, production of the (+) sense single-stranded genomic (49S) and subgenomic (26S) RNAs continues much longer, with the 49S and 26S RNAs ultimately becoming the predominant virus-specific RNA species present in cells infected with an alphavirus [47]. The 49S and 26S RNAs possess 5′ -m^7^G cap structures, 3′ -poly(A) tails, and are completely dependent on the cellular machinery for translation [48]. However, despite these similarities with cellular mRNAs, we showed that the majority of piRNA-like viral small RNAs in mosquito cells were generated from the 49S and 26S mRNAs, suggesting the involvement of a pathogen-associated molecular pattern (PAMP) in the piRNA-like viral small RNA biogenesis pathway.

**Figure 5:**
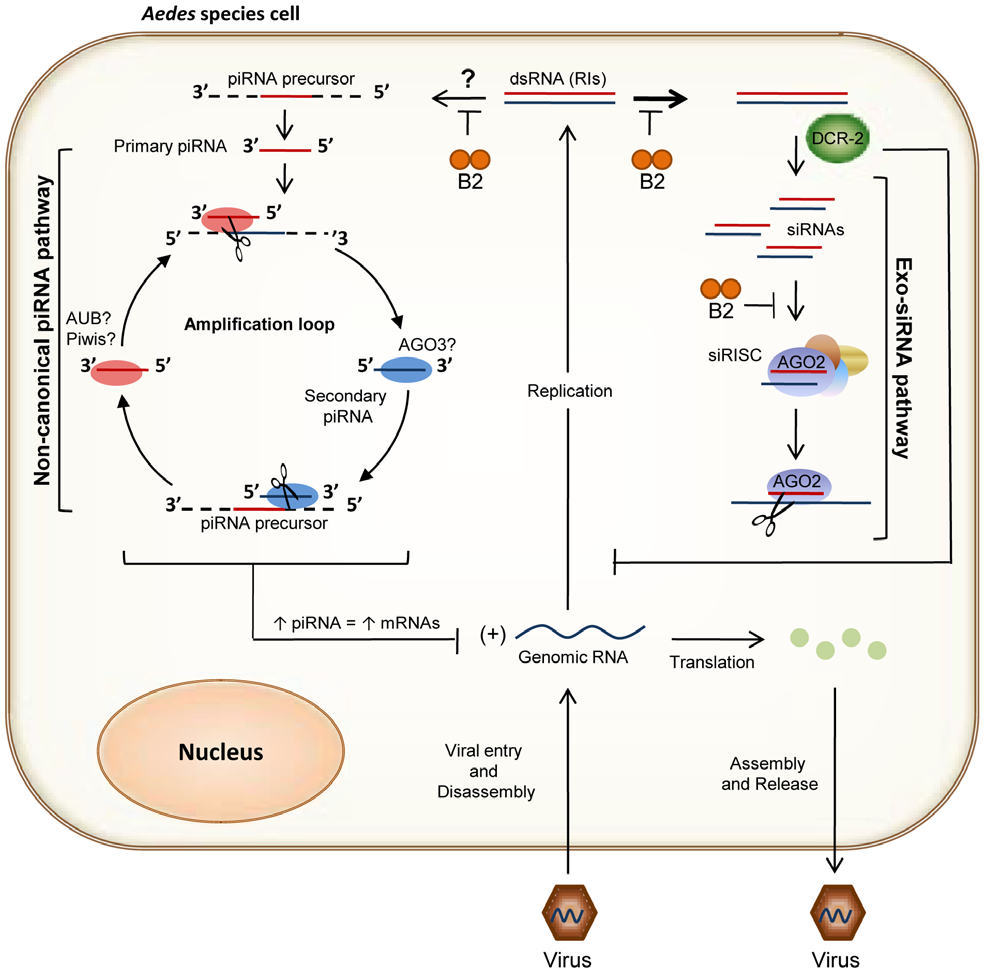
Model for RNA-based immune pathways modulating alphavirus pathogenesis in the mosquito soma. Following entry and uncoating, the genomic (+) strand RNA of an alphavirus serves both as mRNA and as a template for the synthesis of complementary (−) strand RNA. The viral (−) strands then serve as templates for the synthesis of new genomic-length (+) strand RNAs, as well as for shorter subgenomic (+) strand RNAs (26S mRNA) that encode the virus' structural genes. Alphaviruses are thought to synthesize (−) strand RNAs for a limited duration of time early in the infection, establishing an upper limit on the number of dsRNA RIs present in the cell. However, production of the (+) single-stranded genomic (49S) and subgenomic (26S) RNAs continues much longer, ultimately becoming the predominant virus-specific RNAs present in the cell. In this model antiviral siRNA and piRNA-like viral small RNA biogenesis pathways compete for a limited number of precursor dsRNA RIs in the infected cell. While recognition of dsRNA activates both pathways, secondary piRNA-like viral small RNAs are preferentially generated from viral mRNAs. Efficient processing of dsRNA RIs by Dcr-2 may restrict the amount of precursor substrate available to enter the piRNA-like viral small RNA biogenesis pathway. The B2 protein binds both siRNA duplexes and long dsRNAs preventing the protein components of antiviral pathways access to dsRNAs, but inhibition is not absolute. Elevated levels of viral replication may increase amplification of secondary piRNA-like viral small RNAs from 49S and 26S mRNA substrates.

The innate immune systems of insects rely on ancient and well conserved signal transduction pathways to mount an effective response to microbial pathogens. The immune receptors that activate these pathways have evolved the ability to distinguish pathogen from self by recognizing a limited number of broadly conserved PAMPs essential to the survival of the invading microbe but not found in the host. Pattern recognition molecules include lipopolysaccharides, teichoic acids, mannans, and viral dsRNA. It is not uncommon for some of these molecules to activate multiple signal transduction pathways in the infected host. Dependence of multiple small RNA immune pathways on a common dsRNA precursor substrate suggests a mechanism for the coordination of otherwise compartmentalized responses to viral infection. For example, under conditions in which dsRNA is efficiently processed by Dcr-2, relatively small quantities of precursor substrate may be available to the biogenesis pathway for piRNA-like viral small RNAs. Nevertheless, even low levels of precursor substrate entering this pathway may augment a primary antiviral response directed by siRNAs, through ping-pong amplification of secondary piRNA-like viral small RNAs from viral mRNA. However, the modulation of alphavirus pathogenesis observed in *dcr-2* null mutant cells by an antiviral response that appears to be solely directed by piRNA-like viral small RNAs suggests this normally subordinate pathway is capable of providing a redundant antiviral activity in the absence of viral siRNA production. Thus, we propose a model (Figure 5) with many similarities to the hierarchical action of antiviral defenses in some plants, in which there is precedent for functional-redundancy in RNA-based immune responses, but in those cases the redundant antiviral activity being provided by a duplication of Dicer-like proteins [49]. Validation of our model will require specific loss-of-function mutants not currently available for the requisite mosquito species. However, as techniques for the manipulation of animal genomes with zinc finger nucleases (ZFNs) continue to mature, these mutants may soon become available.

To counteract RNA silencing immune pathways, the genomes of many plant and insect viruses have evolved to encode proteins that suppress RNA silencing [50]. This suggests that these virulence factors confer an evolutionary advantage to the pathogen. However, it remains unclear if mosquito-borne viruses encode similar proteins. Redundancy in the antiviral defenses of plants often elicits the production of different sized siRNAs when infected with viruses [51]. For example, siRNAs that are predominately 21-nt in length are generated after infection with some viruses, while infection with other viruses induces predominately 22-nt or 24-nt siRNAs, while still other viruses give rise to a mixture of size classes [51]. This appears to result from the targeting of specific antiviral responses in the plant by various viral suppressor proteins. To counteract this targeting by virus-encoded suppressor proteins, plants appear to have evolved redundant antiviral defenses. It will be interesting to see if the same selection pressure has led to the evolution of redundant antiviral defenses in the mosquito. Characterizing the virus-derived small RNAs generated in response to infection with other mosquito-borne viral pathogens may provide some insight into whether or not these viruses also encode suppressors of RNA-based immune responses. Indeed, previous studies characterizing virus-derived small RNAs in mosquito cells infected with other mosquito-borne viruses already suggest differences with the virus-derived small RNAs described here [52]–[54]. Notably, despite descriptions of larger size classes (24–30 nt) of virus-derived small RNAs, ping-pong-dependent piRNA-like viral small RNAs were not identified by those studies [52]–[54]. It will be interesting to see if the larger classes of virus-derived small RNAs described in those studies are products of a different biogenic pathway than that which gives rise to the piRNA-like viral small RNAs described here.

## Materials and Methods

### Cell culture

We cultured C6/36, u4.4, and CCL-125 cells in DMEM supplemented with FBS. C7-10 cells were cultured as described in [55]. Aag2 cells were cultured in Schneider's Drosophila medium (Lonza) supplemented with FBS. We cultured all cells at 28°C and 5% CO_2_.

### Recombinant virus production and infections

We created p3′dsCHIK by inserting a duplicate subgenomic promoter and multiple cloning site (MCS) immediately downstream of the E1 structural protein coding sequence of pCHIKic. Heterologous sequences were inserted into the MCS of p3′dsCHIK. Recombinant viruses were rescued as described [26]. One to two-day-old female *A. aegypti* (Liverpool) and *A. albopictus* (Wise) were injected in the thorax with 10^4^ pfu of virus. Cells were infected at a multiplicity of infection (MOI) of 0.05.

### RNA isolation and detection

We extracted RNA with Tri Reagent RT (Molecular Research Center). We performed northern blotting with a DNA fragment corresponding to the CHIKV 3′ UTR that we ^32^P-labeled using the Amersham Megaprime DNA Labeling System (GE Healthcare). We analyzed CHIKV (+) strand RNA levels using strand-specific quantitative real-time PCR (ssqPCR) Taqman assay (Life Technologies) as described previously [56], with the following forward (F) and reverse (R) primers and Taqman probe: F: 5′-ATCACAATTGGCAACGAGAAGAG-3′, R: 5′-CTGTGGGTTCGGAGAATAGTGG-3′, Probe: 5′-CTAAAAGCAGCCGAACTC-3′. A total of three biological replicates were used in each ssqPCR assay. A total of four technical replicates were also run on each RNA sample in the assay. A *t-*test was used for significance testing of ssqPCR results.

### Small RNA library preparation, deep sequencing and analysis

We prepared libraries from total RNA isolated from cultured mosquito cells and adult female mosquitoes four days after infection with CHIKV. Libraries were prepared with Illumina's small RNA sample prep kit, according to the manufacturer's instructions, with only minor modifications. In brief, we recovered small RNAs that were 18 to 35-nt in size by PAGE. Single libraries were sequenced on an Illumina GAII. Biological replicate libraries (n = 3) were prepared with Illumina's TruSeq small RNA sample prep kit, multiplexed and sequenced in a single lane of a HiSeq flow cell in order to reduce non-biological sources of variation. After the removal of adaptor sequences and non-coding RNA sequences (rRNAs, tRNAs, snRNAs, snoRNAs, etc.) processed reads were mapped to the CHIKV genome (strain 37997) using Bowtie [57] (v-mode, permitting 1 mismatch). Mappable small RNA reads (vsRNAs, miRNAs, TE-derived small RNAs, endo-siRNAs) were normalized with the TMM method [58] as implemented by the edgeR software package (http://www.bioconductor.org/). As there is no published genome for *A. albopictus,* only vsRNAs and miRNAs were mappable in cells and mosquitoes of this species. The A–C statistic [59] was used to determine differential expression between normalized individual read counts from single libraries. The edgeR software package was used to determine differential expression between data sets with replicates. Raw sequence counts with more than five copies per million reads were used as the input. Weblogo 3 (http://weblogo.threeplusone.com/) plots were generated to analyze the frequency of nucleotide usage in small RNA reads of an identical size class [60].

### Cloning, sequencing and analysis

We synthesized cDNA from total RNA isolated from cultured mosquito cells and adult mosquitoes using RT-PCR and an oligo-dT primer. We amplified the cDNA products of the RT-PCR reactions using PCR and *dcr-2* specific primers. Overlapping amplicons were assembled into complete clones of the *dcr-2* mRNAs. We generated a consensus for the transcripts in each sample by comparing the sequences of three complete *dcr-2* clones. Consensus sequences for the *A. albopictus* and C6/36 *dcr-2* transcripts were further augmented with deep sequencing reads generated from 454 and mRNA-Seq whole transcriptome analysis. After combining reads from all sources the *A. albopictus* and C6/36 *dcr-2* transcripts were sequenced at minimum 10x coverage. We genotyped regions of C6/36 and C7-10 genomic DNA suspected of containing indels using PCR and *dcr-2* specific primers. The homozygosity of the deletions was confirmed by comparing the sequences in 24 clones (12 per cell line) derived from the amplified DNA products. We performed 5′ and 3′ RACE on *dcr-2* transcripts from *A. albopictus* and C6/36 cells using the First Choice RLM-RACE kit (Ambion) according to the manufacturer's instructions. We verified 5′ and 3′ ends using the sequences of the cloned RACE products. Sequencing of the *A. albopictus dcr-2* RACE products revealed two different mRNA isoforms with alternative 5′ UTR sequences. We identified and compared protein domains in Dcr-2 sequences using the Pfam protein database [61] (e-value of 1×10^−9^).

### Accession numbers

High throughput sequencing data sets have been deposited with NCBI's Gene Expression Omnibus under the following accession number: GSE32247. Dicer-2 sequences have been deposited with NCBI under the following accession numbers: JF819820, JF819821, JF819822, JF819823, JF819824.

## Supporting Information

Figure S1 **Replication of CHIKV in mosquitoes.** Northern blot detection of 49S genomic and 26S subgenomic viral RNA in mosquitoes injected with CHIKV (**A**). Virus accumulation in mosquitoes injected with CHIKV (**B**). Example of a piwi-like RNA sequence pair with a 10-nt offset in head and thorax of *A. albopictus* infected with CHIKV (**C**).
(TIF)

Figure S2 **Expression of virus-derived small RNAs in continuous mosquito cell lines.** Size distribution, density plots, and nucleotide analysis of virus-derived small RNAs in mosquito cell lines infected with CHIKV.
(TIF)

Figure S3 **B2-mediated suppression of piRNA-like viral small RNAs in** ***dcr-2*** **null mutant cells.** Size distribution and nucleotide analysis of virus-derived small RNAs in *dcr-2^FS−1^* (C6/36) cells infected with recombinant viruses expressing FHV B2, FHV B2 (C44Y) or FHV B2 (R54Q) (**A, B, and C**)**.** CHIKV (+) strands per virus-derived small RNA in 1ug of total RNA (calculated from normalized 25–29 nt reads identified in the corresponding library) (**D**). Time course of cytopathology in *dcr-2^del 33^* (C7-10) cells infected with recombinant CHIK viruses (20X magnification) (**E**).
(TIF)

Table S1 **Normalized counts of sequenced virus-derived small RNAs.** Virus-derived small RNAs by size, normalized using the trimmed mean of M value (TMM) applied to sequence counts.
(DOC)

Text S1 **Assembly of small RNAs to examine virus populations in cultured mosquito cell lines.**
(DOC)
