# Supplementary figures and images for: Production of Virus-Derived Ping-Pong-Dependent piRNA-like Small RNAs in the Mosquito Soma
Source: PLoS Pathog. 2012 Jan 5;8(1):e1002470. doi: 10.1371/journal.ppat.1002470 (PMC3252369; doi:10.1371/journal.ppat.1002470)

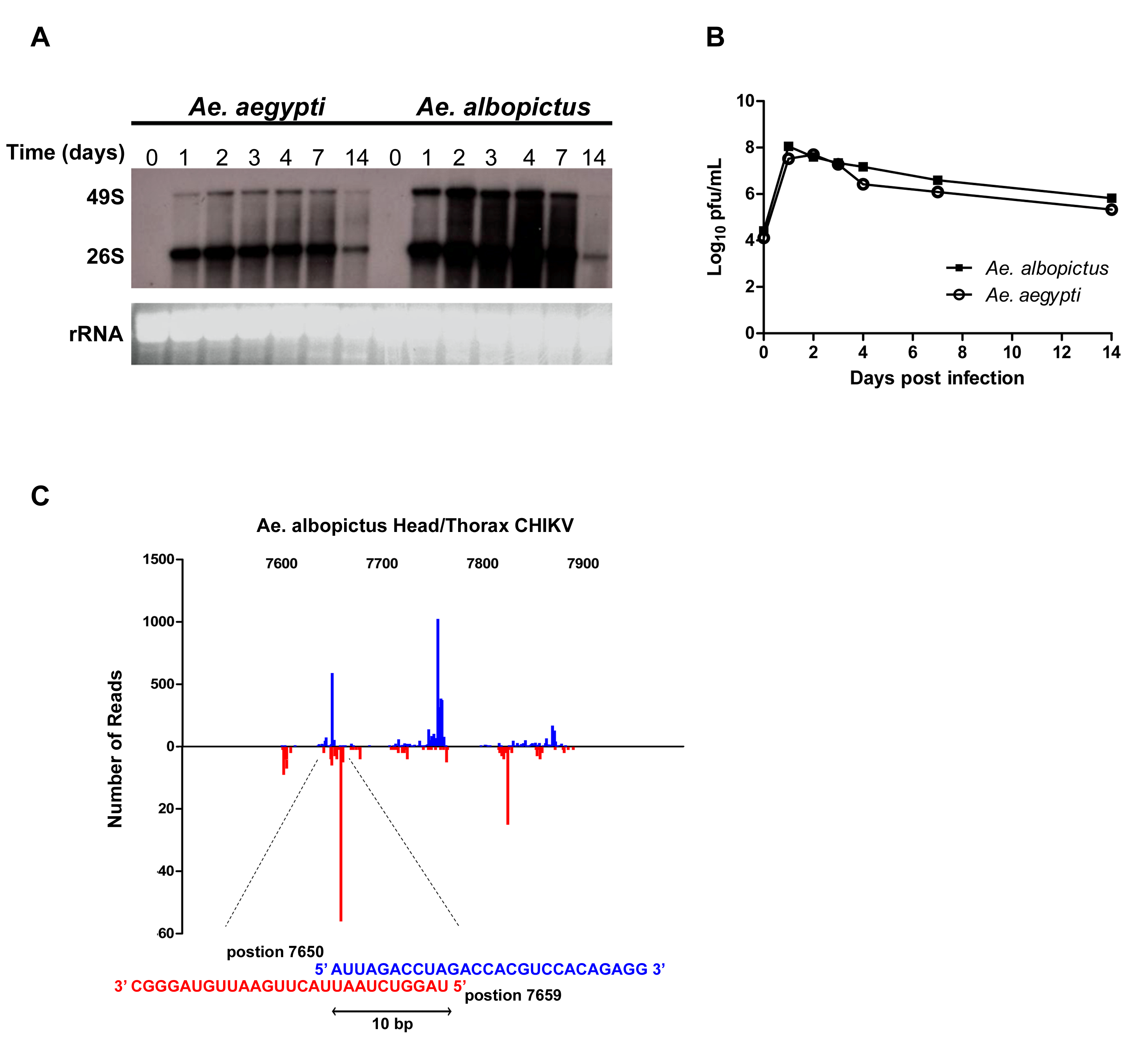

Supplement: Figure S1 — Replication of CHIKV in mosquitoes. Northern blot detection of 49S genomic and 26S subgenomic viral RNA in mosquitoes injected with CHIKV (A). Virus accumulation in mosquitoes injected with CHIKV (B). Example of a piwi-like RNA sequence pair with a 10-nt offset in head and thorax of A. albopictus infected with CHIKV (C). (TIF) [file ppat.1002470.s001.tif]

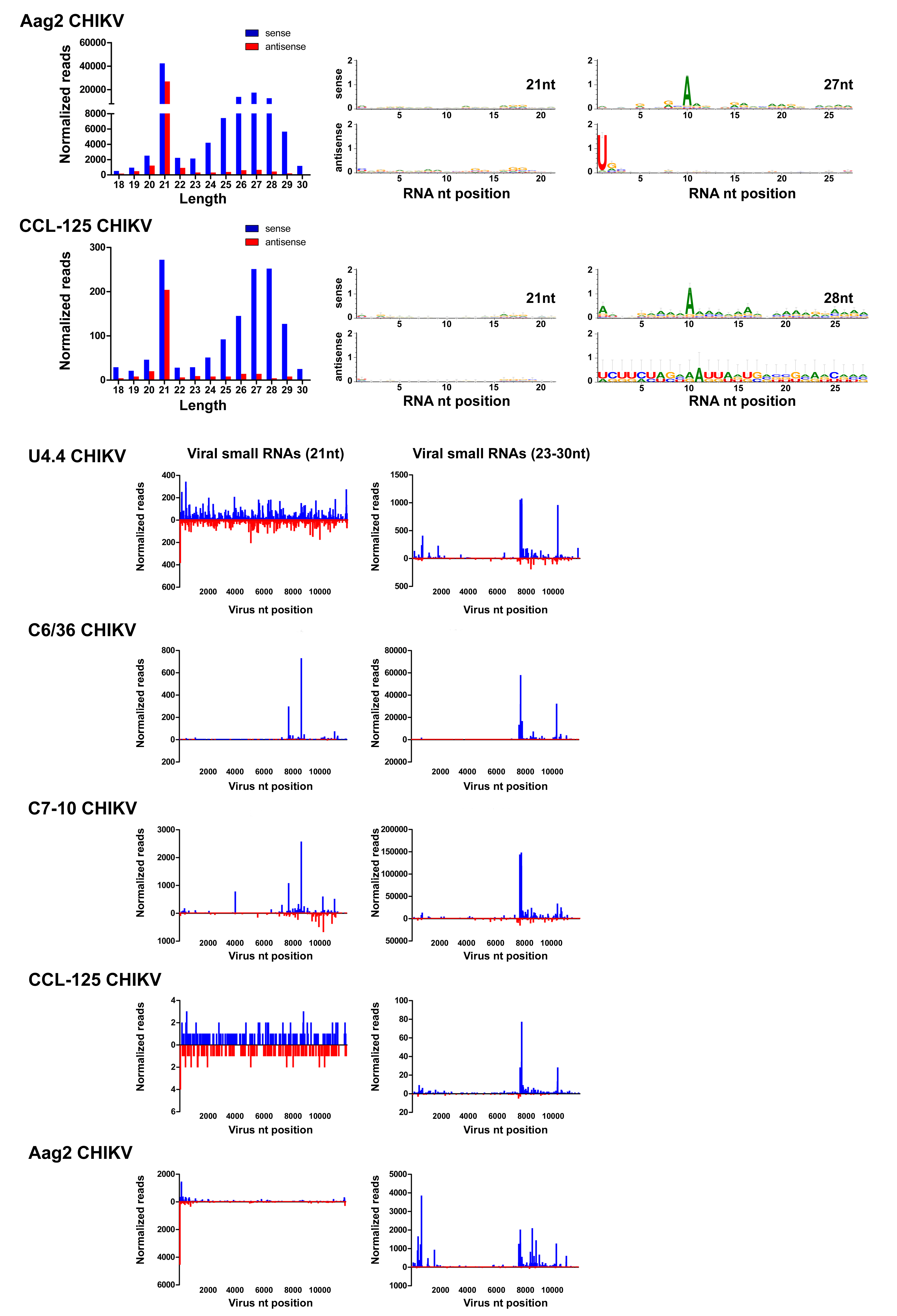

Supplement: Figure S2 — Expression of virus-derived small RNAs in continuous mosquito cell lines. Size distribution, density plots, and nucleotide analysis of virus-derived small RNAs in mosquito cell lines infected with CHIKV. (TIF) [file ppat.1002470.s002.tif]

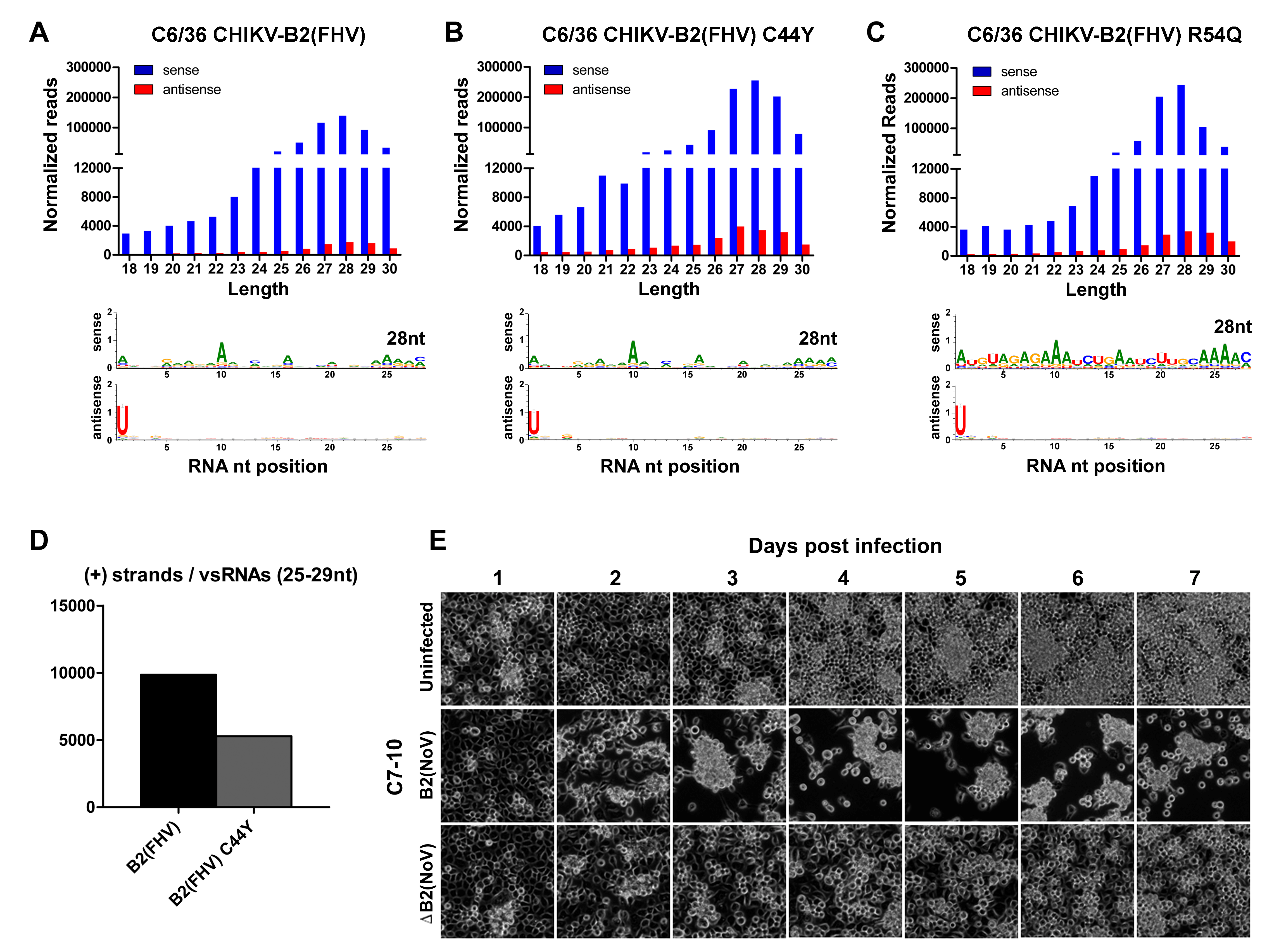

Supplement: Figure S3 — B2-mediated suppression of piRNA-like viral small RNAs in dcr-2 null mutant cells. Size distribution and nucleotide analysis of virus-derived small RNAs in dcr-2FS−1 (C6/36) cells infected with recombinant viruses expressing FHV B2, FHV B2 (C44Y) or FHV B2 (R54Q) (A, B, and C). CHIKV (+) strands per virus-derived small RNA in 1ug of total RNA (calculated from normalized 25–29 nt reads identified in the corresponding library) (D). Time course of cytopathology in dcr-2del 33 (C7-10) cells infected with recombinant CHIK viruses (20X magnification) (E). (TIF) [file ppat.1002470.s003.tif]
