# Supplementary material for: Production of Virus-Derived Ping-Pong-Dependent piRNA-like Small RNAs in the Mosquito Soma
Source: PLoS Pathog. 2012 Jan 5;8(1):e1002470. doi: 10.1371/journal.ppat.1002470 (PMC3252369; doi:10.1371/journal.ppat.1002470)
Supplement: Table S1 — Normalized counts of sequenced virus-derived small RNAs. Virus-derived small RNAs by size, normalized using the trimmed mean of M value (TMM) applied to sequence counts. (DOC) [file ppat.1002470.s004.doc]

| ***Ae. aegypti* WB CHIKV** | **18** | **19** | **20** | **21** | **22** | **23** | **24** | **25** | **26** | **27** | **28** | **29** | **30** |
| --- | --- | --- | --- | --- | --- | --- | --- | --- | --- | --- | --- | --- | --- |
| **Sense vRNAs** | 117 | 272 | 940 | 21547 | 836 | 409 | 254 | 273 | 300 | 486 | 405 | 250 | 84 |
| **Antisense vRNAs** | 36 | 129 | 413 | 10783 | 420 | 155 | 53 | 33 | 30 | 38 | 33 | 18 | 6 |

| ***Ae. albopictus* WB CHIKV** | **18** | **19** | **20** | **21** | **22** | **23** | **24** | **25** | **26** | **27** | **28** | **29** | **30** |
| --- | --- | --- | --- | --- | --- | --- | --- | --- | --- | --- | --- | --- | --- |
| **Sense vRNAs** | 204 | 514 | 2229 | 48835 | 1090 | 483 | 443 | 572 | 822 | 1568 | 1639 | 934 | 187 |
| **Antisense vRNAs** | 120 | 295 | 1013 | 23713 | 611 | 168 | 59 | 80 | 59 | 124 | 84 | 58 | 25 |

| ***Ae. albopictus* HT CHIKV** | **18** | **19** | **20** | **21** | **22** | **23** | **24** | **25** | **26** | **27** | **28** | **29** | **30** |
| --- | --- | --- | --- | --- | --- | --- | --- | --- | --- | --- | --- | --- | --- |
| **Sense vRNAs** | 2343 | 4878 | 16369 | 305530 | 9928 | 3856 | 3185 | 3600 | 5348 | 9580 | 7526 | 3248 | 549 |
| **Antisense vRNAs** | 1139 | 2994 | 9634 | 188615 | 5920 | 1333 | 628 | 556 | 571 | 749 | 523 | 261 | 59 |

***Ae. albopictus* HT**

| **CHIKV B2 (NoV)** | **18** | **19** | **20** | **21** | **22** | **23** | **24** | **25** | **26** | **27** | **28** | **29** | **30** |
| --- | --- | --- | --- | --- | --- | --- | --- | --- | --- | --- | --- | --- | --- |
| **Sense vRNAs** | 8826 | 10634 | 10926 | 78366 | 5389 | 4838 | 5357 | 6862 | 12576 | 24682 | 13763 | 3758 | 453 |
| **Antisense vRNAs** | 3864 | 4102 | 4694 | 42116 | 1334 | 573 | 342 | 353 | 407 | 530 | 241 | 120 | 23 |

***Ae. albopictus* HT**

| **CHIKV ∆B2** | **18** | **19** | **20** | **21** | **22** | **23** | **24** | **25** | **26** | **27** | **28** | **29** | **30** |
| --- | --- | --- | --- | --- | --- | --- | --- | --- | --- | --- | --- | --- | --- |
| **Sense vRNAs** | 3017 | 4953 | 15006 | 329419 | 7337 | 2363 | 2104 | 2158 | 3119 | 5783 | 3919 | 1754 | 256 |
| **Antisense vRNAs** | 2671 | 4814 | 11528 | 243544 | 5166 | 1172 | 628 | 535 | 548 | 693 | 447 | 191 | 52 |

***Ae. albopictus*** HT

| **CHIKV B2 (FHV)** | **18** | **19** | **20** | **21** | **22** | **23** | **24** | **25** | **26** | **27** | **28** | **29** | **30** |
| --- | --- | --- | --- | --- | --- | --- | --- | --- | --- | --- | --- | --- | --- |
| **Sense vRNAs** | 8527 | 11844 | 18273 | 228737 | 6855 | 4572 | 5857 | 7114 | 14385 | 36087 | 35682 | 28236 | 15694 |
| **Antisense vRNAs** | 5184 | 7003 | 11040 | 152257 | 2589 | 858 | 649 | 645 | 700 | 1091 | 1145 | 1060 | 805 |

| **C6/36 CHIKV** | **18** | **19** | **20** | **21** | **22** | **23** | **24** | **25** | **26** | **27** | **28** | **29** | **30** |
| --- | --- | --- | --- | --- | --- | --- | --- | --- | --- | --- | --- | --- | --- |
| **Sense vRNAs** | 638 | 1135 | 1068 | 1665 | 2063 | 4106 | 7665 | 12249 | 30200 | 74295 | 98275 | 86946 | 45946 |
| **Antisense vRNAs** | 23 | 38 | 48 | 62 | 89 | 139 | 213 | 234 | 411 | 620 | 621 | 605 | 364 |

| **C7-10 CHIKV** | **18** | **19** | **20** | **21** | **22** | **23** | **24** | **25** | **26** | **27** | **28** | **29** | **30** |
| --- | --- | --- | --- | --- | --- | --- | --- | --- | --- | --- | --- | --- | --- |
| **Sense vRNAs** | 2676 | 4167 | 6849 | 12116 | 19978 | 37468 | 63583 | 87066 | 153550 | 348716 | 426876 | 304043 | 90623 |
| **Antisense vRNAs** | 1436 | 2825 | 4190 | 6256 | 10885 | 14915 | 21983 | 24503 | 39579 | 49404 | 43793 | 30682 | 9102 |

| **U4.4 CHIKV** | **18** | **19** | **20** | **21** | **22** | **23** | **24** | **25** | **26** | **27** | **28** | **29** | **30** |
| --- | --- | --- | --- | --- | --- | --- | --- | --- | --- | --- | --- | --- | --- |
| **Sense vRNAs** | 1102 | 2264 | 9495 | 109965 | 3658 | 1783 | 2120 | 3376 | 6842 | 14454 | 12361 | 5359 | 954 |
| **Antisense vRNAs** | 872 | 1832 | 6046 | 72970 | 2276 | 861 | 461 | 609 | 823 | 1170 | 844 | 432 | 144 |

| **Aag2 CHIKV** | **18** | **19** | **20** | **21** | **22** | **23** | **24** | **25** | **26** | **27** | **28** | **29** | **30** |
| --- | --- | --- | --- | --- | --- | --- | --- | --- | --- | --- | --- | --- | --- |
| **Sense vRNAs** | 515 | 943 | 2511 | 42368 | 2229 | 2144 | 4207 | 7426 | 13884 | 17513 | 12725 | 5651 | 1180 |
| **Antisense vRNAs** | 165 | 490 | 1221 | 26975 | 922 | 315 | 305 | 377 | 615 | 650 | 443 | 191 | 68 |

| **CCL-125 CHIKV** | **18** | **19** | **20** | **21** | **22** | **23** | **24** | **25** | **26** | **27** | **28** | **29** | **30** |
| --- | --- | --- | --- | --- | --- | --- | --- | --- | --- | --- | --- | --- | --- |
| **Sense vRNAs** | 29 | 21 | 46 | 272 | 28 | 29 | 51 | 92 | 145 | 251 | 252 | 127 | 25 |
| **Antisense vRNAs** | 4 | 8 | 20 | 204 | 6 | 9 | 8 | 8 | 14 | 14 | 4 | 8 | 1 |

| **C6/36 CHIKV B2 (FHV)** | **18** | **19** | **20** | **21** | **22** | **23** | **24** | **25** | **26** | **27** | **28** | **29** | **30** |
| --- | --- | --- | --- | --- | --- | --- | --- | --- | --- | --- | --- | --- | --- |
| **Sense vRNAs** | 2952 | 3326 | 4023 | 4661 | 5271 | 8028 | 12221 | 20451 | 50138 | 116140 | 139623 | 91958 | 33238 |
| **Antisense vRNAs** | 112 | 122 | 204 | 252 | 268 | 403 | 408 | 526 | 830 | 1487 | 1767 | 1634 | 890 |

| **C6/36 CHIKV B2(FHV)C44Y** | **18** | **19** | **20** | **21** | **22** | **23** | **24** | **25** | **26** | **27** | **28** | **29** | **30** |
| --- | --- | --- | --- | --- | --- | --- | --- | --- | --- | --- | --- | --- | --- |
| **Sense vRNAs** | 4082 | 5594 | 6650 | 10984 | 9895 | 18039 | 24227 | 43069 | 91104 | 227671 | 255058 | 202142 | 78613 |
| **Antisense vRNAs** | 478 | 474 | 540 | 753 | 888 | 1068 | 1345 | 1471 | 2411 | 3989 | 3477 | 3199 | 1503 |

| **C6/36 CHIKV B2(FHV)R54Q** | **18** | **19** | **20** | **21** | **22** | **23** | **24** | **25** | **26** | **27** | **28** | **29** | **30** |
| --- | --- | --- | --- | --- | --- | --- | --- | --- | --- | --- | --- | --- | --- |
| **Sense vRNAs** | 3624 | 4112 | 3628 | 4279 | 4816 | 6862 | 11044 | 19337 | 58279 | 204614 | 243625 | 104036 | 38792 |
| **Antisense vRNAs** | 233 | 257 | 280 | 340 | 505 | 672 | 775 | 912 | 1468 | 2922 | 3386 | 3209 | 1991 |

**C6/36 CHIKV B2 (FHV)**

| **Replicate 1** | **18** | **19** | **20** | **21** | **22** | **23** | **24** | **25** | **26** | **27** | **28** | **29** | **30** |
| --- | --- | --- | --- | --- | --- | --- | --- | --- | --- | --- | --- | --- | --- |
| **Sense vRNAs** | 4154 | 4049 | 3706 | 3297 | 3415 | 4518 | 3927 | 5057 | 11988 | 67894 | 21906 | 14963 | 5194 |
| **Antisense vRNAs** | 124 | 179 | 426 | 850 | 934 | 939 | 1177 | 1419 | 1270 | 1545 | 1372 | 1551 | 1110 |

**C6/36 CHIKV B2 (FHV)**

| **Replicate 2** | **18** | **19** | **20** | **21** | **22** | **23** | **24** | **25** | **26** | **27** | **28** | **29** | **30** |
| --- | --- | --- | --- | --- | --- | --- | --- | --- | --- | --- | --- | --- | --- |
| **Sense vRNAs** | 3601 | 3557 | 3104 | 2718 | 2958 | 3725 | 3476 | 4152 | 10649 | 58974 | 18770 | 12854 | 4012 |
| **Antisense vRNAs** | 96 | 127 | 304 | 535 | 739 | 802 | 927 | 1070 | 1060 | 1222 | 1225 | 1230 | 936 |

**C6/36 CHIKV B2 (FHV)**

| **Replicate 3** | **18** | **19** | **20** | **21** | **22** | **23** | **24** | **25** | **26** | **27** | **28** | **29** | **30** |
| --- | --- | --- | --- | --- | --- | --- | --- | --- | --- | --- | --- | --- | --- |
| **Sense vRNAs** | 3383 | 3644 | 3093 | 2476 | 2737 | 3743 | 3190 | 3855 | 10266 | 62473 | 12577 | 6257 | 1186 |
| **Antisense vRNAs** | 131 | 148 | 364 | 615 | 832 | 866 | 1065 | 1164 | 1190 | 1251 | 1123 | 776 | 213 |

**C6/36 CHIKV B2 (FHV) C44A**

| **Replicate 1** | **18** | **19** | **20** | **21** | **22** | **23** | **24** | **25** | **26** | **27** | **28** | **29** | **30** |
| --- | --- | --- | --- | --- | --- | --- | --- | --- | --- | --- | --- | --- | --- |
| **Sense vRNAs** | 3394 | 3225 | 4141 | 3623 | 5323 | 6809 | 6767 | 8601 | 21863 | 118416 | 32553 | 21810 | 7987 |
| **Antisense vRNAs** | 120 | 145 | 268 | 379 | 708 | 843 | 1113 | 2715 | 2909 | 3449 | 2633 | 2659 | 1898 |

**C6/36 CHIKV B2 (FHV) C44A**

| **Replicate 2** | **18** | **19** | **20** | **21** | **22** | **23** | **24** | **25** | **26** | **27** | **28** | **29** | **30** |
| --- | --- | --- | --- | --- | --- | --- | --- | --- | --- | --- | --- | --- | --- |
| **Sense vRNAs** | 3432 | 3199 | 3496 | 3254 | 4725 | 6210 | 5829 | 6758 | 16949 | 85131 | 28892 | 18264 | 6668 |
| **Antisense vRNAs** | 113 | 118 | 199 | 266 | 568 | 555 | 800 | 1961 | 2481 | 2493 | 2225 | 1938 | 1578 |

**C6/36 CHIKV B2 (FHV) C44A**

| **Replicate 3** | **18** | **19** | **20** | **21** | **22** | **23** | **24** | **25** | **26** | **27** | **28** | **29** | **30** |
| --- | --- | --- | --- | --- | --- | --- | --- | --- | --- | --- | --- | --- | --- |
| **Sense vRNAs** | 3342 | 3229 | 3735 | 3108 | 4479 | 5103 | 5180 | 6256 | 15857 | 83597 | 26949 | 17362 | 6085 |
| **Antisense vRNAs** | 93 | 145 | 179 | 327 | 529 | 592 | 770 | 1849 | 2106 | 2438 | 1928 | 1911 | 1445 |
