# Supplementary material for: Production of Virus-Derived Ping-Pong-Dependent piRNA-like Small RNAs in the Mosquito Soma
Source: PLoS Pathog. 2012 Jan 5;8(1):e1002470. doi: 10.1371/journal.ppat.1002470 (PMC3252369; doi:10.1371/journal.ppat.1002470)
Supplement: Text S1 — Assembly of small RNAs to examine virus populations in cultured mosquito cell lines. (DOC) [file ppat.1002470.s005.doc]

The ability to assemble cloned viral siRNA sequences into long contiguous fragments has provided a novel approach to virus discovery by deep sequencing . We utilized this approach to determine if cultured mosquito cells and adult mosquitoes had any established persistent viral infections. For assembly of small RNA reads we used the Velvet program with a k-mer of 17 and a minimum contig length of 100 as described previously . To identify virus-specific contigs we searched the non-redundant protein databases of the National Center for Biotechnology Information (NCBI) by BLASTX. Our results revealed mix infection of several mosquito cell lines by viruses of various genome types. Analysis of small RNA sequence contigs confirm a persistent infection of C6/36 and Aag2 cells by C6/36 densovirus and cell fusion agent virus, respectively . A large percentage of small RNA sequence contigs from u4.4 cells also exhibited partial identity with Drosophila X virus (DXV) suggesting these cells are persistently infected with a previously unidentified member of the *Birnaviridae*. Other than those of CHIKV, virus-specific contigs were not identified in the C7-10 and CCL-125 cell lines, or in *A. albopictus* and *A. aegypti*.

1. Wu Q, Luo Y, Lu R, Lau N, Lai EC, et al. Virus discovery by deep sequencing and assembly of virus-derived small silencing RNAs. Proc Natl Acad Sci U S A 107: 1606-1611.

2. Zerbino DR, Birney E (2008) Velvet: algorithms for de novo short read assembly using de Bruijn graphs. Genome Res 18: 821-829.

3. Chen S, Cheng L, Zhang Q, Lin W, Lu X, et al. (2004) Genetic, biochemical, and structural characterization of a new densovirus isolated from a chronically infected Aedes albopictus C6/36 cell line. Virology 318: 123-133.

4. Scott JC, Brackney DE, Campbell CL, Bondu-Hawkins V, Hjelle B, et al. (2010) Comparison of Dengue Virus Type 2-Specific Small RNAs from RNA Interference-Competent and -Incompetent Mosquito Cells. PLoS Negl Trop Dis 4: e848.
